# Disseminated TB in an immunosuppressed patient with previous negative QuantiFERON

**DOI:** 10.1177/11206721261428920

**Published:** 2026-02-27

**Authors:** Jae Yee Ku, Shi Zhuan Tan

**Affiliations:** 1 St Paul's Eye Unit, 105725 Royal Liverpool University Hospital, Liverpool, UK; 2 Department of Eye and Vision Science, Institute of Life Course and Medical Sciences, Faculty of Health & Life Sciences, University of Liverpool, Liverpool, UK

## Descriptive caption

A 44-year-old Caucasian woman with Behçet's disease and ulcerative colitis on adalimumab presented with right eye presumed inflammatory panuveitis (A). She showed a limited response to oral prednisolone. Vitreous biopsy revealed *Mycobacterium tuberculosis* (TB). Systemic imaging, including chest radiography (diffuse patchy opacities; B) and MRI head, confirmed disseminated TB. Following discussion at the TB multi-disciplinary team meeting, she was switched to systemic dexamethasone as per the Thwaites protocol, adapted for the management of TB with cerebral involvement in immunocompromised individuals. Two weeks after commencing anti-TB therapy and while on tapering dexamethasone, she developed severe intraocular inflammation (organised hypopyon filling the entire anterior chamber, with overlying superficial vessels; C), which improved after dexamethasone escalation (D). She was systemically well throughout and had a negative QuantiFERON 12 months earlier. This case highlights the consideration of TB in immunosuppressed patients despite a previous negative QuantiFERON and the importance of cautious and gradual tapering of systemic corticosteroids in the first 3 months after commencement of treatment.

**Figure:**
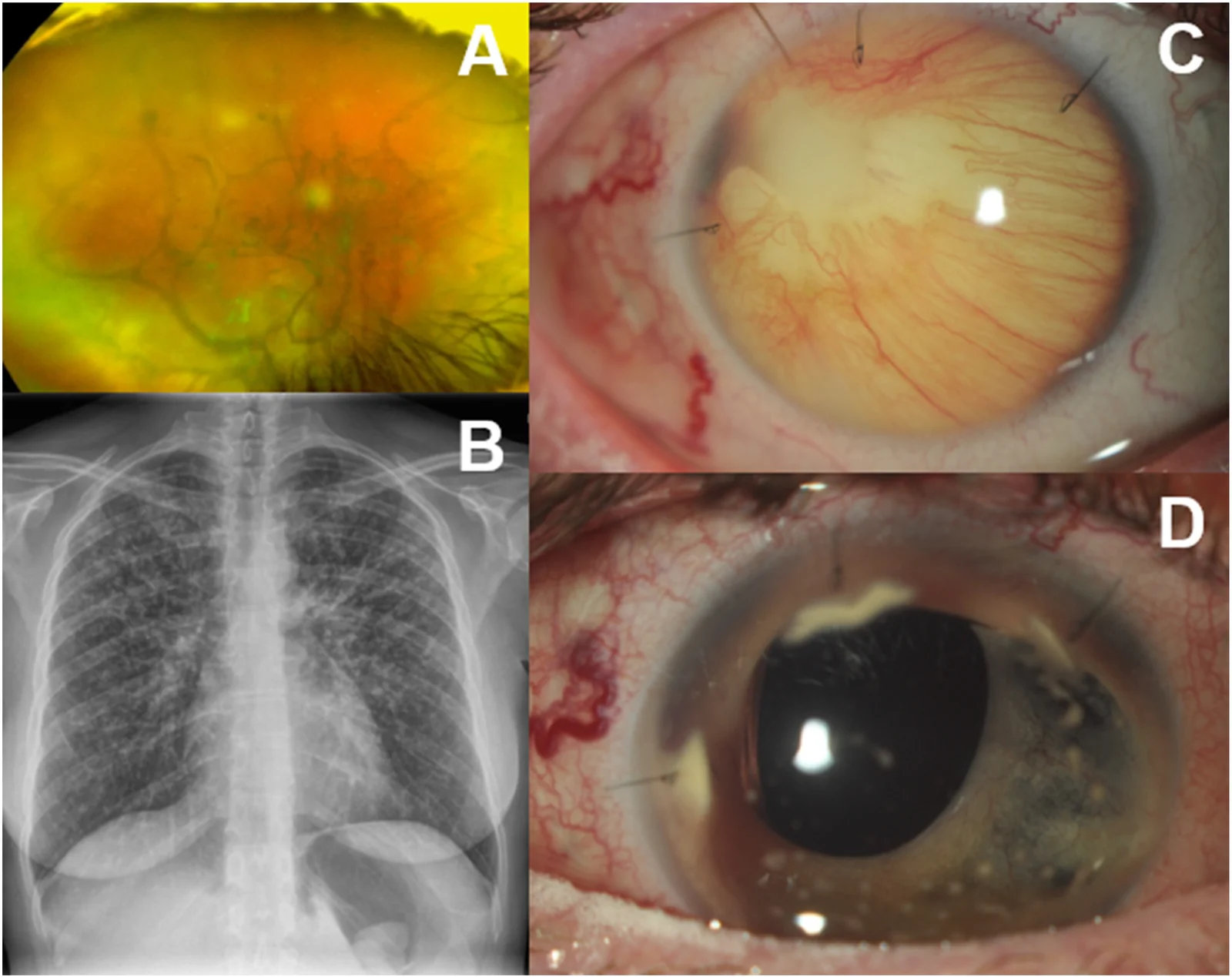


## Consent

Informed consent was obtained from the patient

